# Overexpression of tRNA m^7^G modification methyltransferase complex promotes the biosynthesis of triterpene in yeast

**DOI:** 10.3389/fmicb.2025.1557443

**Published:** 2025-03-31

**Authors:** Mengyu Ma, Jun Wang, Zhengwei Tan, Xiqin Liang, Bengui Fan, Lei Li, Huizhen Liang, Tianyue An, Guoli Wang

**Affiliations:** ^1^Featured Laboratory for Biosynthesis and Target Discovery of Active Components of Traditional Chinese Medicine, School of Traditional Chinese Medicine, Binzhou Medical University, Yantai, China; ^2^Institute of Chinese Herbal Medicines, Henan Academy of Agricultural Sciences, Zhengzhou, China; ^3^Provincial Key Laboratory of Conservation and Utilization of Traditional Chinese Medicine Resources, Institute of Chinese Herbal Medicines, Henan Academy of Agricultural Sciences, Zhengzhou, China

**Keywords:** tRNA m^7^G modification, metabolic engineering, terpenoids, transcriptome analysis, *Saccharomyces cerevisiae*

## Abstract

**Background:**

The sustainable production of valuable compounds using microbial cell factories is an effective approach, yet further metabolic engineering strategies are needed to enhance their biosynthetic potential. Recent studies suggest that RNA modifications can influence cellular metabolism, but their role in metabolic engineering remains largely unexplored.

**Methods:**

The production of squalene and lupeol in different yeast strains was detected by gas chromatography-mass spectrometry (GC-MS) equipment. Transcriptomic analysis was performed to identify metabolic changes associated with the epigenetic modification. The transcriptional and translational expression of targeted genes were determined by real-time quantitative polymerase chain reaction and western blotting, respectively. The mRNA stability of targeted genes was measured by mRNA decay assay.

**Results:**

In this study, the overexpression of Trm8 and Trm82 complex, mediating the tRNA 7-methylguanosine (m^7^G) modification in yeast, significantly increased the production of squalene in CEN.PK2-1C. Transcriptome analysis indicated that Trm8/Trm82 overexpression upregulated the expression levels of genes involved in amino acid synthesis, glycolysis, and tricarboxylic acid cycle, and the enhanced glycolysis, upstream of acetyl-CoA biosynthesis, might be responsible for the promoted biosynthesis of squalene. Further investigation demonstrated that Trm8/Trm82 complex could increase the production of squalene and lupeol in engineered yeast.

**Conclusion:**

These findings indicate that tRNA m^7^G modification can regulate central metabolism and enhance terpenoid biosynthesis. This study provides new insights into RNA modifications as a potential metabolic engineering strategy for improving the production of high-value compounds.

## 1 Introduction

Natural compounds, particularly terpenoids, recognized as the largest family among natural products, exhibit substantial potential for applications within the pharmaceutical, food, fragrance, fuel, and agricultural industries (Beekwilder et al., 2024; Chen et al., 2024; Pan et al., 2024). This potential is primarily attributed to their intricate and distinctive chemical compositions coupled with diverse biological activities. Terpenoids serve as essential sources in the development of new drugs, displaying an extensive range of pharmacological effects. For instance, compounds like ajmalicine, taxol, and rare ginsenosides demonstrate a variety of bioactivities, including antiarrhythmic, antitumor, antimicrobial, anti-inflammatory, and hypoglycemic properties (Zhu and Chen, 2019; Li M. et al., 2023; Guo et al., 2024). Furthermore, terpenoids such as menthol, citronellol, and carvacrol are widely applied within the fragrance and food industries (Harwood et al., 2022; Ma et al., 2024). Currently, the procurement of natural compounds largely depends on direct plant extraction and chemical synthesis. However, each of these approaches presents certain constraints (Jiang and Wang, 2023). Direct extraction generally necessitates plant materials, which are limited by climate and growth cycles, thus resulting in low product yields. On the other hand, chemical synthesis involves intricate procedures and may lead to environmental contamination. Due to these limitations associated with conventional preparation methods, biosynthesis has garnered extensive attention as an environmentally benign and sustainable alternative approach.

The successful elucidation of the biosynthetic pathways of important natural products, such as taxol, bergenin and asiaticoside (Jiang et al., 2024; Yan et al., 2024; Zhao et al., 2024), promote the application of heterologous synthesis strategies grounded in metabolic engineering for the efficient production of these complex chemicals. Implementing synthetic biology strategies to develop microbial cell factories for the sustainable production of valuable compounds in *Escherichia coli* and yeast has proven to be a highly effective approach (Paramasivan and Mutturi, 2022; Li Y. et al., 2023). By regulating the expression of triacylglycerides in lipid droplets and the squalene synthase Erg9 protein, metabolic flux within the carotenoid pathway was increased while minimizing *β*-carotene-induced stress on cell membranes. This adjustment led to 107.3% and 49.5% enhancements in β-carotene production relative to the original strain (Bu et al., 2022). In *Saccharomyces cerevisiae*, the catalytic activity of *α*-santalene synthase was optimized through semi-rational protein engineering, and the storage capacity for α-santalene was expanded by overexpressing the DGA1 gene, which is involved in TAG biosynthesis. This approach yielded a shake-flask production of 213.7 ± 12.4 mg/L α-santalene in the engineered yeast strain (Yu et al., 2020). A platform integrating compatible heterologous enzymes and iterative metabolic engineering screening enabled the identification and screening of key cytochrome P450 enzymes essential for ganoderic acid synthesis. By optimizing the catalytic sequence of cytochrome P450 enzymes, ganoderic acid production in yeast ultimately surpassed that achieved in *Ganoderma lucidum* (Yuan et al., 2022). Currently, mainstream metabolic engineering strategies include the construction and optimization of metabolic pathways, identification and modification of key enzymes, cofactor regeneration engineering, and enhancement of cellular tolerance (Wu et al., 2024). Nonetheless, as the production of target compounds reaches certain levels, the benefits of these strategies may become constrained. Consequently, exploring and innovating new metabolic engineering strategies is essential to further augment the production potential of microbial cell factories.

In recent years, RNA modifications have garnered increasing attention. Among these, the 7-methylguanosine (m^7^G) modification is a prevalent form, primarily located in tRNA, rRNA, and mRNA, where it has significant impacts on RNA stability, translation efficiency, and function (Li R. et al., 2023). The tRNA m^7^G modification, widely present across eukaryotes, bacteria, and some archaea, was first identified in yeast and is notable as one of the few modifications that result in positively charged bases under physiological conditions (Matsumoto et al., 2007; Weng et al., 2023; Zhu et al., 2023). As a positively charged RNA modification, m^7^G serves essential roles in gene expression, metabolic processing, protein synthesis, and transcriptional stability (Cole and Cowling, 2009; Xia et al., 2023). This modification occurs at position 46 within the variable loop of tRNA and is catalyzed by the Trm8/Trm82 complex in yeast and the METTL1/WDR4 complex in human cells, providing structural stability to the tertiary structure of tRNA (Malbec et al., 2019). The m^7^G modification in tRNA is particularly crucial in the rapid tRNA decay pathway and also influences mRNA translation (Alexandrov et al., 2006; Fu et al., 2024). Currently, research on the functions of tRNA m^7^G remains limited. In *S. cerevisiae*, the tRNA m^7^G methyltransferase complex comprises Trm8 and Trm82 (Alexandrov et al., 2002a). In this study, overexpression of the Trm8/Trm82 complex in yeast cells was found to promote squalene biosynthesis. Transcriptome sequencing indicated that Trm8/Trm82 overexpression led to enhanced expression of numerous genes associated with amino acid synthesis, glycolysis, and the tricarboxylic acid cycle pathways. Further investigation demonstrated that the overexpression of the Trm8/Trm82 complex could increase yields of squalene and lupeol in engineered yeast, suggesting the potential application of tRNA m^7^G modification in yeast terpenoid metabolic engineering.

## 2 Materials and methods

### 2.1 Construction of plasmids and strains

Genomic DNA from *S. cerevisiae* strain CEN.PK2-1C was isolated using the FastPure Plant DNA Isolation Mini Kit (Vazyme Biotech, China). Endogenous yeast genes, along with promoters and terminators, were cloned from this extracted genome. The genes *EfMvaS*, *EfMvaE*, and *GgLUS* were synthesized by General Biol (China). The mutation of *EfMvaS^A110G^* was introduced via the Fast Site-Directed Mutagenesis Kit (TianGen Biotech, China). All gene, promoter, and terminator fragments were ligated into the pEASY®-Blunt cloning vector (TransGen Biotech, China) for sequence confirmation, and gene expression cassettes were subsequently assembled through an overlap polymerase chain reaction. All plasmids were constructed with the ClonExpress Ultra One Step Cloning Kit (Vazyme Biotech, China). Yeast expression fragments were amplified from the respective plasmids using 2 × Phanta Max Master Mix (Vazyme Biotech, China) and then integrated into the yeast genome through the PEG/LiAc transformation method. All plasmids and strains constructed in this study are listed in Table 1, and the primers in this part was listed in Supplementary Table S1.

**Table 1:** Plasmids and strains constructed in this work.

Plasmids	Description	Source
pESC-LEU	Yeast expression vector	Agilent Technologies
pESC-URA	Yeast expression vector	Agilent Technologies
pESC-HIS	Yeast expression vector	Agilent Technologies
pESC-TRP	Yeast expression vector	Agilent Technologies
pEASY-Blunt	Gene cloning vector	TransGen Biotech
pSH65	Yeast gene editing vector	Addgene
pESC-KITRP	Replace TRP marker with loxP sites-flanked TRP (KITRP) marker	This study
pESC-KILEU	Replace LEU marker with loxP sites-flanked LEU (KILEU) marker	This study
pESC-KIHIS	Replace HIS marker with loxP sites-flanked HIS (KIHIS) marker	This study
pESC-KIURA	Replace URA marker with loxP sites-flanked URA (KIURA) marker	This study
pMY01	Replace *GAL10p* with *PGK1p* and replace *GAL1p* with *TEF1p* in pESC-KILEU	This study
pMY02	Insert *TRM8* into pMT01	This study
pMY03	Insert *EfMvaS* into pESC-KITRP	This study
pMY04	Insert *EfMvaE* into pMY03	This study
pMY05	Insert *Erg8* into pESC-KIHIS	This study
pMY06	Insert *ERG12* into pMY05	This study
pMY07	Insert *IDI1* into pESC-URA	This study
pMY08	Insert *ERG19* into pMY07	This study
pMY09	Insert *ERG10* into pESC-HIS	This study
pMY10	Insert *ERG10* expression cassette from pMY09 to pMY08	This study
pMY11	Insert *SmFPS* into pESC-KIURA	This study
pMY12	Insert *AtSQS2* into pMY11	This study
pMY13	Insert *GgLUS1* into pESC-TRP	This study

Strains	Genotype	Source
CEN.PK2-1C	*MATa leu2-3,112 ura3-52 trp1-289 his3-Δ1 MAL2-8c SUC2*	ATCC
MY01	CEN.PK2-1C *TRM82p*:: KILEU *PGK1p-TRM8-ADH1t-TEF1p*	This study
MY02	CEN.PK2-1C *LPP1*:: KITRP *GAL10p*-*EfMvaE*-*ADH1t*-*GAL1p*-*EfMvaS*-*CYC1t*	This study
MY03	MY02 *URA3*:: KIHIS *GAL10p-ERG8-ADH1t-GAL1p*-*ERG12*-*CYC1t*	This study
MY04	MY03 308a:: *GAL10p-IDI1-ADH1t-GAL1p-ERG19-CYC1t-GAL10p-ERG10-ADH1t*	This study
MY05	MY04 *NDT80*:: *LEU GAL10p-SmFPS-ADH1t-GAL1p-AtSQS2-CYC1t*	This Study
MY06	MY05 loxPΔ	This study
MY07	MY06 *TRM82p*:: KILEU *PGK1p-TRM8-ADH1t-TEF1p*	This study
MY08	MY06:: pMY13	This study
MY09	MY07:: pMY13	This study

### 2.2 Shake flask fermentation

The fermentation method was performed according to the previous report (Lin et al., 2023). Briefly, single colony of positive transformants were introduced into a 5 mL SD medium (6.67 g/L yeast nitrogen base without amino acids, 20 g/L glucose, and corresponding amino acids) selective medium and maintained at 30°C with agitation at 220 rpm for a period of 12–16 h. Following this initial incubation, a calculated volume of the resultant culture was transferred to 50 mL of the SD medium to establish an initial optical density of 0.1 at OD_600_. The carbon source was provided as either 5 mL of 20 g/L glucose or an induction carbon source consisting of 2 g/L glucose and 18 g/L galactose. The cultures underwent further incubation at 30°C and 220 rpm for a duration of 120–144 h.

### 2.3 Extraction and detection of compounds

The method for extraction and detection of compounds was adapted to the previous report (Gan et al., 2024). In summary, after fermentation, the yeast culture was subjected to centrifugation at 5,000 rpm for 5 min at 4°C to separate the precipitated yeast cells. The collected cells were then combined with 10 mL of an alkaline lysis solution containing 20% (v/v) potassium hydroxide and 50% (v/v) ethanol, thoroughly vortexed, heated at boiling for 15 min, and subsequently allowed to cool to room temperature. The lysed cells and supernatant were combined, and an equal volume of hexane was added, followed by sonication for 30 min before being left to stand for 48 h. Thereafter, the upper organic phase was evaporated to dryness with a rotary evaporator, treated with 50 μL N-Methyl-N-(trimethylsilyl) trifluoroacetamide at 80°C for 30 min, and analyzed by gas chromatography–mass spectrometry (GC-MS) equipment.

GC-MS detection conditions: Analysis was conducted using a 19091S-433UI GC column in combination with a 5977B single quadrupole MS equipped with an electron ionization source at 70 eV. A solvent delay of 10 min was applied in the MS, with the electron multiplier voltage activated and a gain factor of 1. The injection volume was 1 μL, employed in either splitless mode or with a split ratio of 50:1. The column temperature was initially set to 160°C, maintained for 1 min, then elevated to 280°C at a rate of 30°C/min and held for 10 min, subsequently increased to 300°C at a rate of 2°C/min, and sustained for 5 min.

### 2.4 Construction of standard curves

The squalene and lupeol standards were initially dissolved in ethyl acetate to create stock solutions at a concentration of 1 mg/mL. From these solutions, 100 μL was withdrawn and further diluted to achieve concentrations of 10, 20, 40, 80, and 160 mg/L. A 1 mL aliquot from each of the diluted solutions was then transferred into clean vials and subsequently dried under a stream of nitrogen. The dried samples were derivatized with 50 μL of N-Methyl-N-(trimethylsilyl) trifluoroacetamide at 80°C for 30 min prior to GC-MS analysis.

### 2.5 Real-time quantitative polymerase chain reaction

Total RNA was isolated from yeast employing a yeast total RNA rapid extraction kit and subsequently reverse-transcribed into cDNA utilizing the HiScript II Q RT SuperMix for qPCR kit (Vazyme Biotech, China). UBC6 was designated as the reference gene, and real-time quantitative polymerase chain reaction (RT-qPCR) was conducted with the ChamQ Universal SYBR qPCR Master Mix kit (Vazyme Biotech, China). The primer sequences are listed in Supplementary Table S2.

### 2.6 Transcriptome sequencing

Cells from the CEN.PK2-1C and MY01 strains were harvested during the logarithmic growth phase and subjected to centrifugation at 3,000 rpm at 4°C. Total RNA was isolated using Trizol reagent (Invitrogen), with RNA concentration and quality evaluated using a NanoDrop 2000 spectrophotometer (Thermo Fisher, China). Subsequently, RNA integrity was verified with the Agilent RNA Nano 6000 kit. mRNA was purified from total RNA through Thermo Fisher Scientific Dynabeads™ Oligo(dT)25 magnetic beads, and sequencing was conducted on the Illumina Novaseq platform by Novogene (China). Differential gene expression analysis was carried out with the DEseq2 method, and the identified differentially expressed genes (DEGs) underwent Gene Ontology (GO) and Kyoto Encyclopedia of Genes and Genomes (KEGG) enrichment analysis.

### 2.7 Western blotting

To detect the protein expression of targeted protein, Flag or Myc tag was fused to the N terminal of targeted protein. The protein tag transformed strains were cultivated and the total proteins were extracted by Yeast Total Protein Extraction Kit (Sangon Biotech). Then the protein extracts were separated by SDS-PAGE and hatched with corresponding antibodies against Flag (Abmart, M20008S) or Myc (Abmart, M20002S), and HSP70 (Abmart, YHA2100) overnight. After incubation with corresponding secondary antibodies for 1 h, the immunoreactive bands were observed by using Tanon-5200 imaging system (Tanon). The densities of targeted bands were measured by using Image J (NIH). HSP70 was used as the reference protein here.

### 2.8 mRNA decay assay

The yeast strains of CEN.PK2-1C and MY01 were cultured to the concentration of OD600 = 0.5 in YPD medium with glucose as the carbon source and a final concentration of 5 μg/L thiolutin was added to the culture. Samples were taken at different times after thiolutin addition. Then RNAs were isolated and qPCR was performed to detect the expression of targeted genes and the reference gene RDN25-1 by using primers listed in Supplementary Table S2.

### 2.9 Statistical analysis

Data processing and statistical analysis were conducted through GraphPad Prism 8 software. All experiments were executed in triplicate, with results expressed as mean ± standard deviation (M ± SD).

## 3 Results

### 3.1 Overexpression of tRNA m^7^G methyltransferase complex Trm8/Trm82 enhanced squalene biosynthesis in yeast

**Figure 1:**
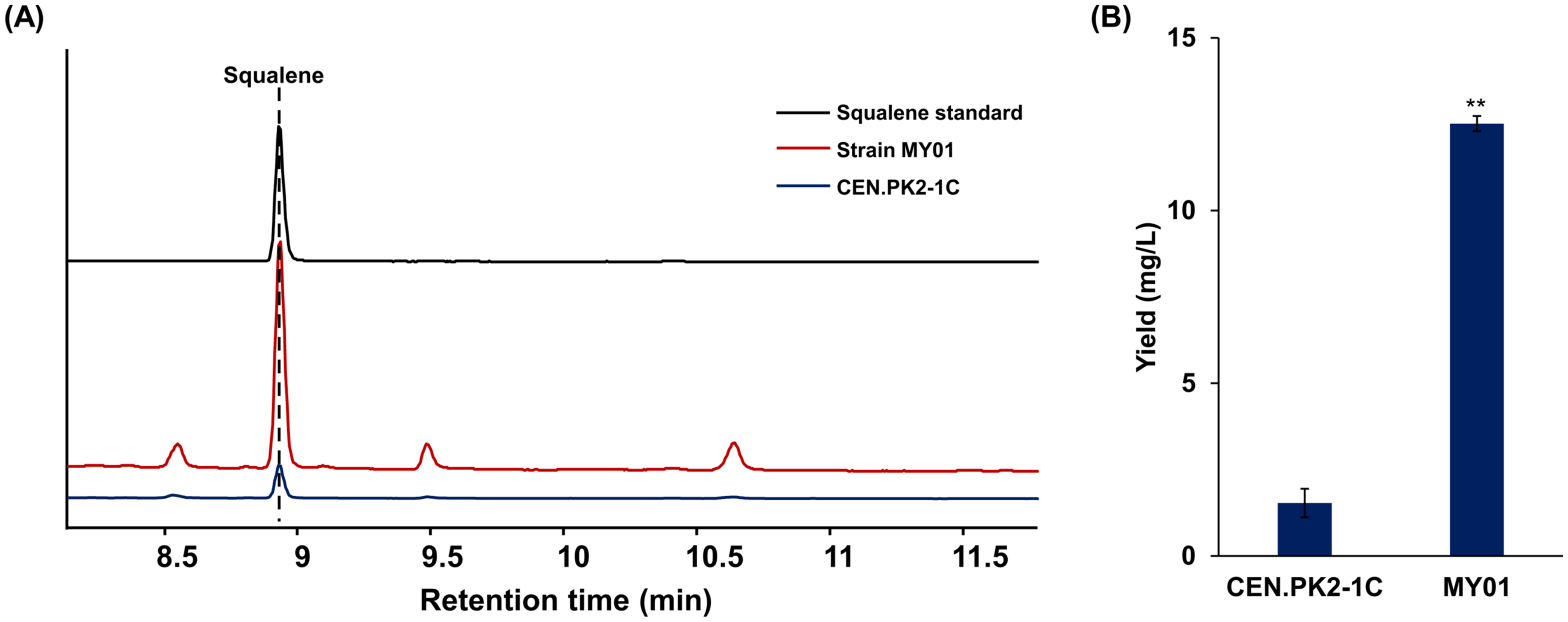
The overexpression of Trm8/Trm82 promoted squalene biosynthesis. **(A)** GC-MS detection of triterpene in different yeast strains. **(B)** The production of squalene in different strains. Strain MY01 was the yeast strain with Trm8/Trm82 overexpression. The asterisks indicate significant differences (^**^p < 0.01, ^*^p < 0.05).

In yeast, the Trm8/Trm82 function as the tRNA m^7^G methyltransferase complex responsible for catalyzing tRNA m^7^G modification and participating in biological processes (BP) such as pre-mRNA splicing and RNA export. The absence of either subunit disrupts the activity of tRNA m^7^G methyltransferase (Alexandrov et al., 2002b; Chen et al., 2022). Prior research has demonstrated that deletion of Trm8, Trm82, or both does not influence yeast growth or development. To investigate the regulatory function of this complex in yeast, both genes were simultaneously overexpressed in the *S. cerevisiae* strain CEN.PK2-1C, and their expression level was confirmed by RT-qPCR and WB (Supplementary Figure S1). And subsequent analyses of metabolite profiles were performed. Growth curve analysis indicated that the Trm82/Trm8 overexpressing strain MY01 exhibited enhanced growth advantage during the cultivation process from logarithm stage to plateau stage (Supplementary Figure S2). Metabolite analysis, quantified using squalene standard curves (Supplementary Figure S3), revealed that the squalene content in strain MY01 was increased (Figure 1A; Supplementary Figure S4) and reached 12.52 ± 0.22 mg/L, representing an 8.2-fold increase relative to the wild type (Figure 1B). Intriguingly, individual deletion of Trm8 or Trm82 reduced squalene production, whereas double deletion increased it by 4.5-fold relative to control (Supplementary Figure S5). Among these strains, Trm8/Trm82 overexpression achieved the highest squalene enhancement, indicating its critical role in boosting triterpene biosynthesis in yeast.

### 3.2 Trm8/Trm82 overexpression markedly regulates genes involved in central carbon metabolism and amino acid synthesis

**Figure 2:**
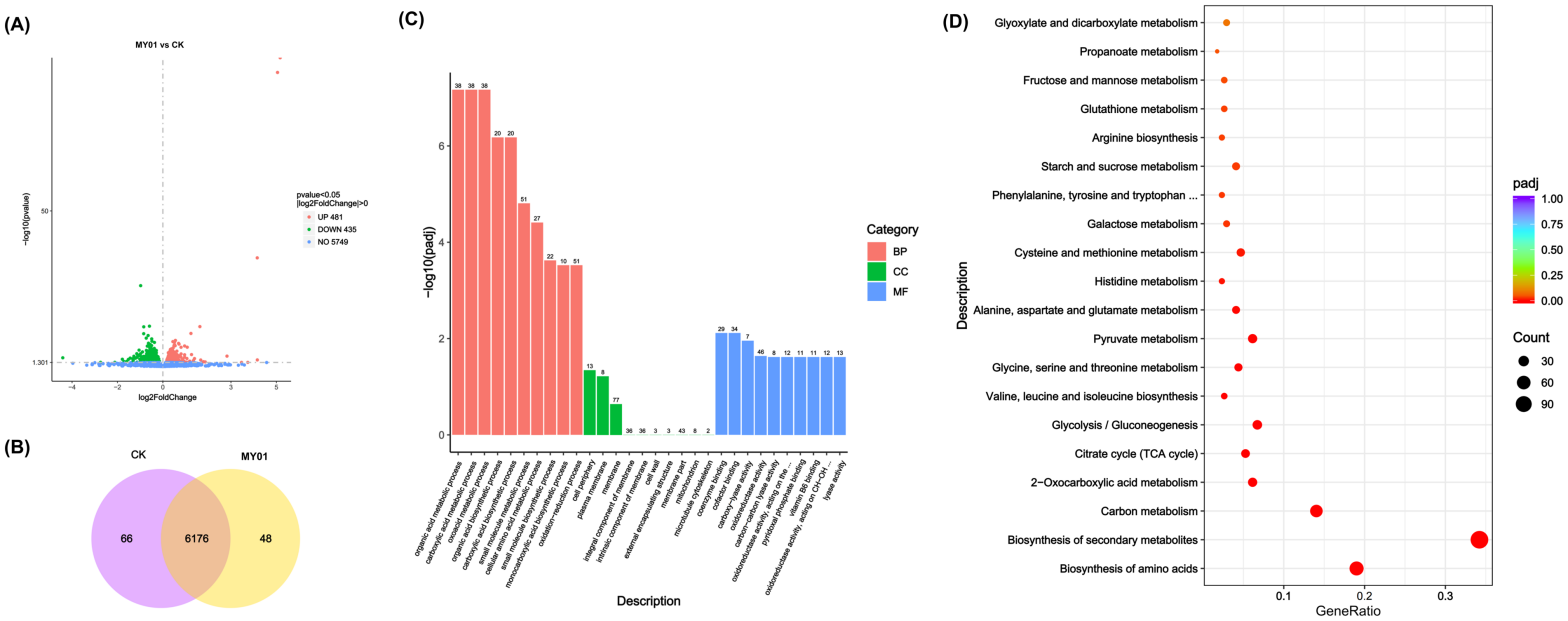
Transcriptome analysis of Trm-TRm82 overexpressed strain. **(A)** The Volcano plot of DEGs in different strains. **(B)** Venn diagram of DEGs in different strains. **(C)** GO analysis of DEGs in different yeast strains. **(D)** KEGG analysis of DEGs in different yeast strains.

To further examine the regulatory effects of the Trm8/Trm82 complex on yeast, transcriptome sequencing analysis was conducted on the MY01 strain and the control strain. The results identified 1,016 DEGs between the MY01 and the control strains, with 481 upregulated and 435 downregulated (Figure 2A). Additionally, strain-specific expression profiles showed 48 genes unique to MY01 versus 66 in wild-type (Figure 2B). GO enrichment analysis indicated that these DEGs were predominantly associated with the organic acid metabolic process, carboxylic acid metabolic process, and oxoacid metabolic process for BP; cell periphery and plasma membrane for cellular component (CC); and coenzyme binding and cofactor binding for molecular function (MF) (Figure 2C), suggesting that TRM82 and TRM8 primarily influenced metabolic processes. KEGG enrichment analysis revealed that the DEGs were chiefly enriched in pathways related to amino acid biosynthesis, secondary metabolite biosynthesis, carbon metabolism, 2-oxocarboxylic acid metabolism, the tricarboxylic acid cycle (TCA cycle), and glycolysis/gluconeogenesis (Figure 2D), suggesting coordinated regulation of primary and secondary metabolism by Trm8/Trm82.

**Figure 3:**
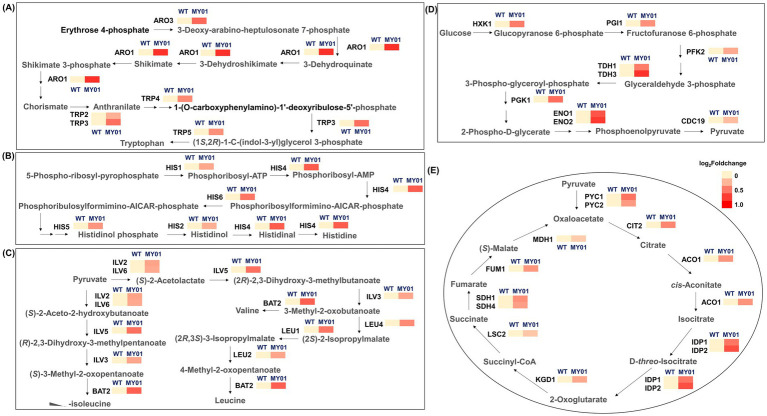
The expression of genes involved in different metabolic pathways. **(A)** The expression of genes involved in tryptophan biosynthesis. ARO3, 3-deoxy-arabine-heptulosonate-7-phosphate synthase; ARO1, 3-dehydroquinate synthase; TRP2, anthranilate synthase; TRP3, indole-3-glycerol phosphate synthase; TRP4, anthranilate phosphoribosyl transferase; TRP5, tryptophan synthetase. **(B)** The expression of genes involved in histidine biosynthesis. HIS1, ATP phosphoribosyltransferase; HIS2, histidinolphosphatase; HIS4, histidinol dehydrogenase; HIS5, histidinol-phosphate aminotransferase; HIS6, histidinol-phosphate aminotransferase. **(C)** The expression of genes involved in valine, leucine and isoleucine biosynthesis. ILV2, acetolactate synthase; ILV3, dihydroxy-acid dehydratase; ILV5, acetohydroxyacid reductoisomerase; ILV6, acetolactate synthase; BAT2, branched-chain amino acid transaminase; LEU1, isopropylmalate isomerase; LEU4, alpha-isopropylmalate synthase. **(D)** The expression of genes involved in glycolysis. HXK1, hexokinase; PGI1, glucose-6-phosphate isomerase; PFK2, phosphofructokinase; TDH1/3, glyceraldehyde-3-phosphate dehydrogenase; PGK1, 3-phosphoglycerate kinase; ENO1/2, enolase; CDC19, pyruvate kinase. **(E)** The expression of genes involved in TCA cycle. PYC1/2, pyruvate carboxylase; CIT2, citrate synthase; ACO1, aconitase; IDP2, NADP-dependent isocitrate dehydrogenase; KGD1, 2-ketoglutarate dehydrogenase; LSC2, succinyl-CoA ligase; SDH1/4, succinate dehydrogenase; FUM1, succinate dehydrogenase; MDH1, mitochondrial malate dehydrogenase.

The tRNA m^7^G methyltransferase complex Trm8/Trm82 enhances translational fidelity through structural stabilization of tRNAs, requiring increased amino acid availability for protein synthesis (Dai et al., 2021; Kaneko et al., 2024). Notably, KEGG enrichment analysis revealed that the most markedly enriched differential genes were associated with amino acid biosynthesis (Figure 2D). Consequently, the biosynthesis of various amino acids in strain MY01 was further investigated. The findings demonstrated an increase in the biosynthesis of various amino acids, with upregulation observed in the genes involved in the biosynthetic pathways of tryptophan, tryptophan, valine, leucine, isoleucine (Figures 3A–C). These observations suggest that overexpression of Trm8/Trm82 markedly promoted the biosynthesis of certain amino acids.

Besides amino acid biosynthesis, KEGG enrichment analysis indicated that pathways associated with carbon metabolism were also enriched in the MY01 strain (Figure 2D). Consequently, carbon metabolism pathways, including glycolysis and the TCA cycle, were further examined. Transcriptome analysis revealed that the expression levels of nearly all enzyme-encoding genes involved in glycolysis were upregulated in the MY01 strain (Figure 3D). Similarly, the expression levels of genes encoding enzymes responsible for nearly all steps of the TCA cycle were also upregulated (Figure 3E). These findings suggest that the m^7^G modification mediated by Trm8/Trm82 promoted the enhancement of central carbon metabolism pathways.

### 3.3 Trm8/Trm82 overexpression enhances terpenoid biosynthesis by upregulating the expression of genes involved in the upstream pathway of acetyl-CoA synthesis

Yeast terpenoid biosynthesis initiates from acetyl-CoA through the mevalonate (MVA) pathway, generating isopentenyl pyrophosphate (IPP) and dimethylallyl pyrophosphate (DMAPP) (Chen et al., 2020; Wang et al., 2024). These intermediates then enter the sterol synthesis pathway, where they are catalyzed by farnesyl pyrophosphate synthase (Erg20) and squalene synthase (Erg9) to produce triterpenes such as squalene (Guo et al., 2022). To explore the mechanism underlying the increased triterpene production in strain MY01, transcriptomic analysis was performed on both pathways. The findings indicated that, no significant changes in the expression levels of enzymes involved in MVA and sterol synthesis pathways, suggesting that the terpenoid biosynthesis pathway was not markedly enhanced in strain MY01.

**Figure 4:**
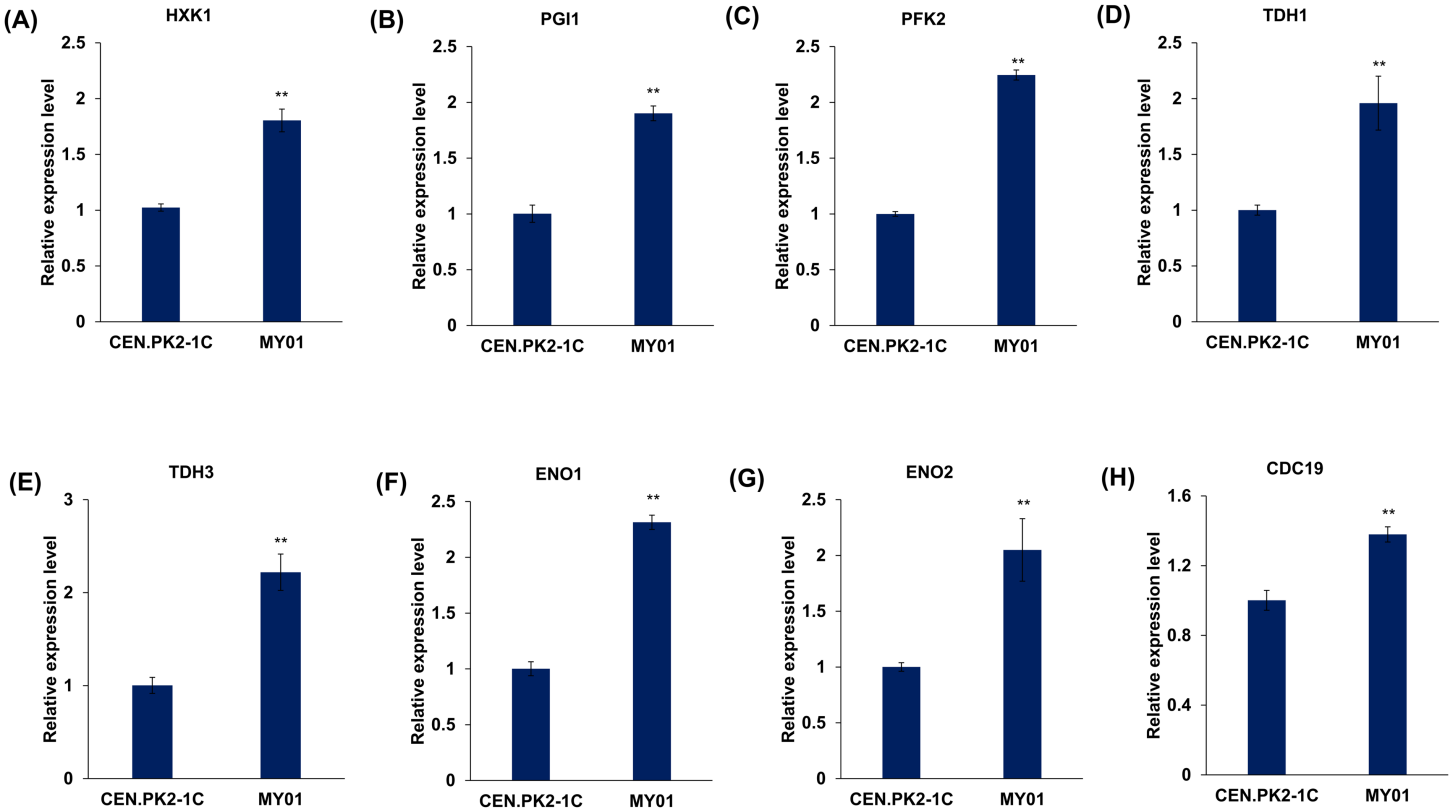
The validation of expression level of genes involved in glycolysis by qRT-PCR. **(A–H)** The expression level of *HXK1*, *PGI1*, *PFK1*, *TDH1*, *TDH3*, *ENO1*, *ENO2* and *CDC19*. The asterisks indicate significant differences (**p < 0.01, *p < 0.05).

Given that MVA substrate acetyl-CoA originates from glycolysis previously shown to be upregulated in MY01 (Figure 3D). To further validate the expression of genes associated with glycolysis, several genes with notably upregulated expression levels, including *HXK1*, *TDH1*, *TDH3*, *PGI1*, *PFK2*, *ENO1*, *ENO2*, and *CDC19*, were chosen for RT-qPCR verification, and four representative genes, including *HXK1*, *PFK2*, *ENO1* and *CDC19*, were chosen for WB verification. The results demonstrated a significant upregulation in the transcriptional and translational expression levels of these genes (Figure 4; Supplementary Figure S6), affirming the enhancement of the glycolytic pathway.

**Figure 5:**
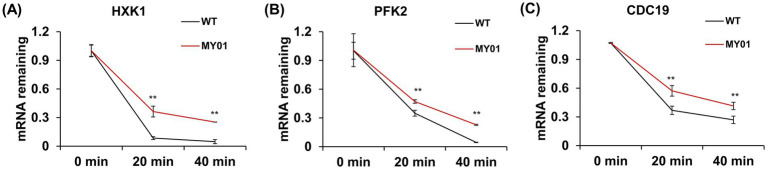
The mRNA decay of selected genes in glycolysis between strain MY01 and the control strain. **(A–C)** The mRNA decay of *HXK1*, *PFK2* and *CDC19*. The asterisks indicate significant differences (**p < 0.01, *p < 0.05).

To further determine the direct role of the upregulated genes in squalene synthesis improvement, we overexpressed the above WB-verified four genes in the control strain, and measured the squalene contents in these strains. The result showed that, Functional characterization through gene overexpression demonstrated that *HXK1*, *ENO1*, and *CDC19* enhanced squalene production, while PFK2 exhibited mild suppression (Supplementary Figure S7). As m^7^G modification was reported to improve mRNA stability, we wondered whether the mRNA stability of the above upregulated genes was enhanced. To verify this, mRNA decay assay was used to detect the mRNA stability of three representative genes in glycolysis, including *HXK1*, *ENO1* and *CDC19*. As shown in Figure 5, the mRNA decay rate of these genes was indeed decelerated compared to the control strain. These observations collectively suggest that Trm8/Trm82 facilitated the synthesis of acetyl-CoA by promoting glycolysis, thereby increasing terpene compound yield.

### 3.4 Trm8/Trm82 overexpression enhanced squalene biosynthesis in engineered strains

**Figure 6:**
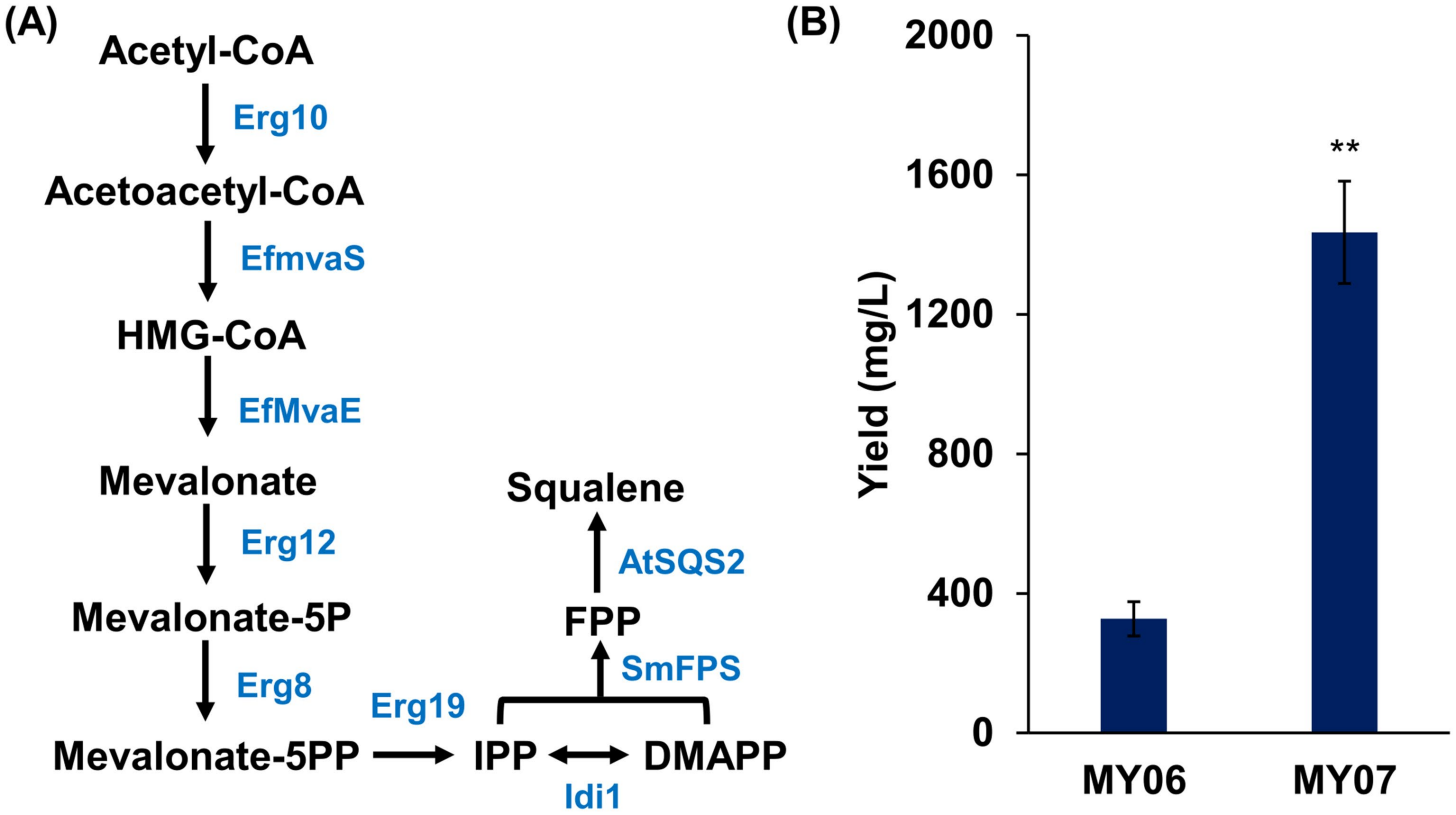
The overexpression of Trm8/Trm82 promoted squalene production in engineered yeast. **(A)** The optimization of squalene biosynthesis pathway. Erg10, acetoacetyl-CoA thiolase; Erg13, HMG-CoA synthase; EfMvaE, HMG-CoA reductase from *Enterococcus faecalis*; EfMvaS, mevalonate synthase from *E. faecalis*; Erg12, mevalonate kinase; Erg8, phosphomevalonate kinase; Erg19, mevalonate pyrophosphate decarboxylase; Idi1, isopentenyl diphosphate: dimethylallyl diphosphate isomerase; SmFPS, farnesyl pyrophosphate synthetase from *Salvia miltiorrhiza*; AtSQS2, squalene synthase from *Arabidopsis thaliana*. **(B)** The squalene production in different yeast strains. The asterisks indicate significant differences (^**^p < 0.01, ^*^p < 0.05).

Trm8/Trm82-mediated m^7^G modification represents a novel metabolic engineering strategy for enhancing yeast triterpene production. To validate this approach, engineered strains with elevated triterpene production were constructed, and Trm8/Trm82 was then overexpressed to assess its effect on the biosynthesis of target compounds by comparing triterpene yields before and after overexpression. For the construction of strains with high terpene production, optimization of the MVA pathway was undertaken initially (Figure 6A). Given that Hmgr serves as a key rate-limiting enzyme in the MVA pathway, EfMvaE from *Enterococcus faecalis* was introduced to enhance this catalytic step. EfMvaE is recognized as the most efficient Hmgr variant for increasing MVA pathway flux, with the capacity to raise squalene levels in yeast strains by up to nine-fold (Lu et al., 2022). Additionally, EfMvaS, an HMG-CoA synthase from *E. faecalis*, demonstrated superior catalytic activity compared to the native yeast Erg13 and was thus introduced to further reinforce the MVA pathway (Dusséaux et al., 2020). Finally, MVA pathway optimization was completed by overexpressing ERG10, ERG12, ERG8, ERG19, and IDI1, resulting in the engineered strain MY04.

Within the sterol synthesis pathway, Erg20 catalyzes the conversion of IPP and DMAPP into farnesyl pyrophosphate (FPP), while two FPP molecules are condensed by Erg9 to form squalene (Guo et al., 2022). To enhance this pathway, the farnesyl diphosphate synthase SmFPS from *Salvia miltiorrhiza* and the squalene synthase AtSQS2 from *Arabidopsis thaliana* were overexpressed in strain MY04 (Wang et al., 2018; Gan et al., 2024), yielding the engineered strain MY05 with elevated triterpene production potential (Figure 6A). Subsequently, Trm8/Trm82 was overexpressed. These two strains were then subjected to shake flask fermentation, followed by triterpene quantification. Based on calculations from the squalene standard curve, strain MY06 produced 327.08 ± 49.15 mg/L of squalene, whereas strain MY07 achieved a squalene yield of 1435.41 ± 146.67 mg/L, marking a 4.4-fold increase compared to strain MY06 (Figure 6B). These findings indicate that enhanced m^7^G modification effectively facilitated squalene biosynthesis in the engineered strain.

### 3.5 Trm8/Trm82 overexpression enhanced the biosynthesis of lupeol in engineered strains

**Figure 7:**
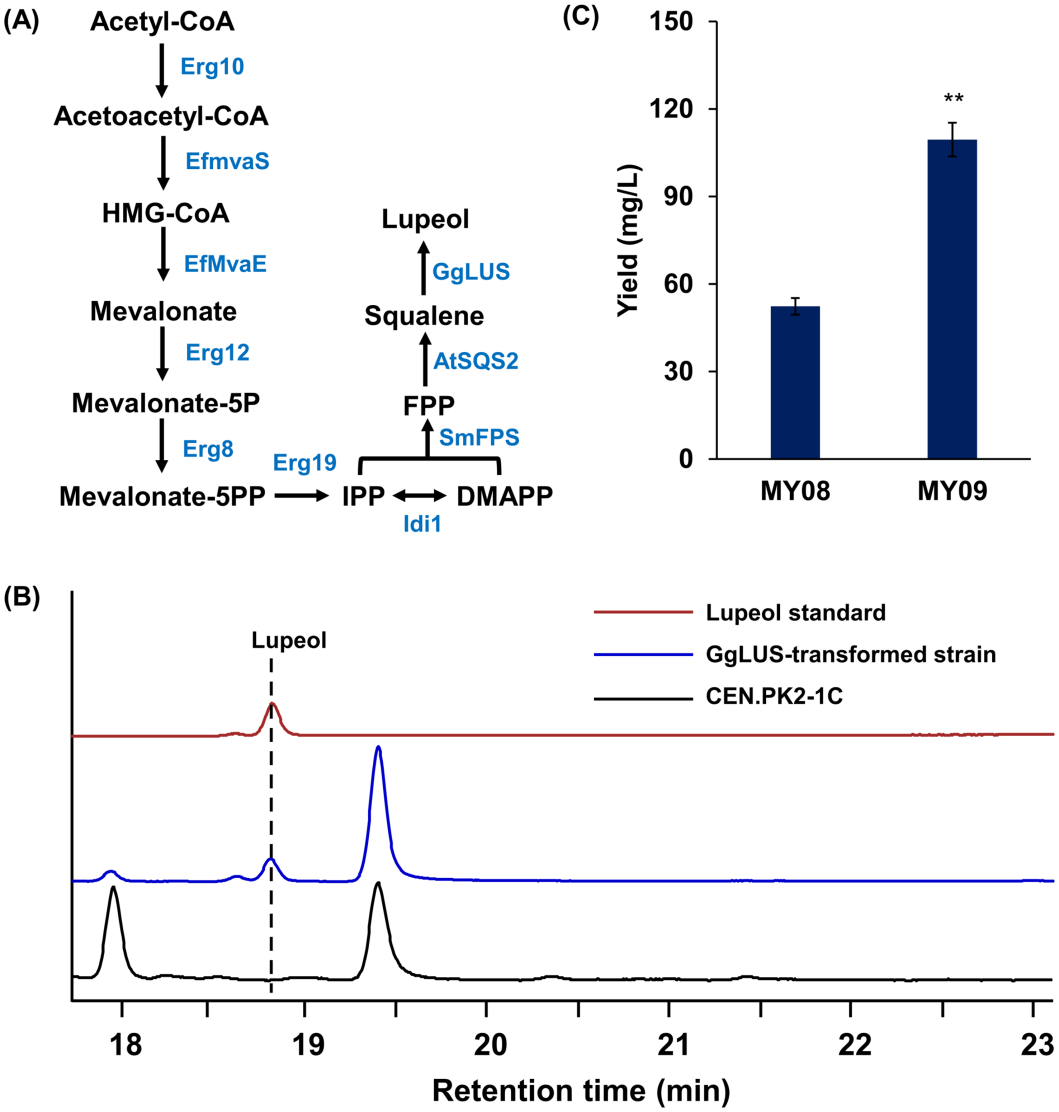
The overexpression of Trm8/Trm82 promoted lupeol production in engineered yeast. **(A)** The optimization of lupeol biosynthesis pathway. Erg10, acetoacetyl-CoA thiolase; Erg13, HMG-CoA synthase; EfMvaE, HMG-CoA reductase from *E. faecalis*; EfMvaS, mevalonate synthase from *E. faecalis*; Erg12, mevalonate kinase; Erg8, phosphomevalonate kinase; Erg19, mevalonate pyrophosphate decarboxylase; Idi1, isopentenyl diphosphate: dimethylallyl diphosphate isomerase; SmFPS, farnesyl pyrophosphate synthetase from *S. miltiorrhiza*; AtSQS2, squalene synthase from *A. thaliana*; GgLUS, codon-optimized lupeol synthase from *Glycyrrhiza glabra.* **(B)** GC-MS detection of lupeol standard and its production in engineered yeast. **(C)** The lupeol production in different yeast strains. The asterisks indicate significant differences (^**^p < 0.01, ^*^p < 0.05).

To further confirm the enhancing effect of m^7^G modification on triterpene biosynthesis, lupeol was selected as a representative triterpene to examine the impact of Trm8/Trm82 on its production. The codon-optimized lupeol synthase (*GgLUS*) from *Glycyrrhiza glabra*, which efficiently catalyzes the formation of lupeol, was introduced into strains MY06 and MY07, resulting in the generation of strains MY08 and MY09, respectively (Figure 7A). Following fermentation, lupeol production was detected and quantified by GC-MS analysis (Figure 7B; Supplementary Figure S8). According to calculations based on the lupeol standard curve (Supplementary Figure S9), the yields of lupeol in strains MY08 and MY09 were 52.34 ± 2.85 mg/L and 109.51 ± 5.78 mg/L, respectively, with enhanced m^7^G modification contributing to a 2.1-fold increase in lupeol production (Figure 7C). These findings demonstrate that Trm8/Trm82 markedly facilitated lupeol biosynthesis in the engineered strains.

## 4 Discussion

The m^7^G methylation modification is a prevalent and essential post-transcriptional modification of RNA molecules, fulfilling critical roles in numerous cellular processes (Chen et al., 2020). The m^7^G methyltransferases have been shown to act as key regulators in a variety of physiological processes, with their dysregulation linked to multiple human diseases. In *S. cerevisiae*, the m^7^G modification, specifically the N7-methylation at position 46 of tRNA, is crucial for tRNA stability and functionality. By influencing protein translation, this modification markedly contributes to the regulation of gene expression and the maintenance of cellular function (Malbec et al., 2019; Li et al., 2024). During meiosis, the regulation of protein synthesis is indispensable for cell cycle progression, and tRNA serves an integral function in this process. While m^6^A methylation has been widely examined in relation to yeast protein synthesis and cell cycle control, research on m^7^G modification, which similarly impacts protein synthesis, remains relatively scarce.

Prior investigations have demonstrated that the deletion of Trm8 or Trm82, or both, does not impact tRNA expression or yeast growth development. In this study, overexpression of Trm8/Trm82 in haploid yeast was found to enhance the yeast growth rate and promote the biosynthesis of triterpene compounds, such as squalene. Gene expression analysis revealed that Trm8/Trm82 overexpression markedly influenced the synthesis of amino acids, including aromatic amino acids, and enhanced central carbon metabolic pathways, such as glycolysis and TCA cycle. Research on m^7^G modification in humans and animals has similarly reported its regulatory effects on cell growth, amino acid synthesis, and metabolic pathways like glycolysis, suggesting a conserved regulatory role of this modification across species.

Significant advancements have been achieved in the construction of *S. cerevisiae* cell factories for the production of high-value natural products through various metabolic engineering strategies. However, numerous challenges persist (Sun et al., 2019). The effectiveness of many current metabolic regulation strategies diminishes once yield reaches a certain threshold. Consequently, the exploration and development of novel metabolic engineering techniques remain essential. In this study, overexpression of the m^7^G methyltransferase complex Trm8/Trm82 was found to promote squalene biosynthesis in *S. cerevisiae*. Further investigation indicated that overexpression of this complex could also substantially enhance the production of triterpenes, such as squalene and lupeol, in highly engineered strains, highlighting the potential of Trm8/Trm82 as an optimization strategy in terpene biosynthesis for metabolic engineering.

Here, the overexpression of Trm8/Trm82 brought about an 8.2-fold increase in squalene production by upregulating the expression of genes involved in glycolysis, the upstream pathway of acetyl-CoA synthesis, in the base strain (Supplementary Figure S10), which presented a significant improvement in squalene biosynthesis compared to the commonly used metabolic engineering approaches. The classic optimization of MVA pathway is the modification of the rate-limiting enzyme Hmgr, and the introduction of the truncated Hmgr (tHMGR) increased the titer of squalene to about 3-fold (Paramasivan and Mutturi, 2017). And the most efficient Hmgr variant EfMvaE could raise the production of squalene to 9-fold higher in yeast (Lu et al., 2022). The combination of overexpression of tHMGR and Erg13, and knockout of ROX1, a negative regulator of MVA pathway, resulted in a significant 8.2-fold increase in the squalene content (Bröker et al., 2018). Another strategy by the improvement of the cofactor NADPH via the overexpression of Zwf1 and Pos5 led to 6.1- and 3.8-fold increase in squalene production, respectively (Paramasivan and Mutturi, 2017). For the biosynthesis of lupeol, the overexpression of Trm8/Trm82 contributed to a 2.1-fold increase in lupeol production in engineered strain, and a previous report have revealed that the extra integration of lupeol synthase into the yeast genome gave a 1.1-fold increase in lupeol production (Tang et al., 2024). The introduction of squalene epoxidase from different host produced 1.6–2.6-fold higher lupeol levels compared to the control strain (Qiao et al., 2019). And a 9.2-fold increase of lupeol production by the optimization of central carbon metabolism was derived in our previous report (Lin et al., 2023). Therefore, the manipulation of Trm8/Trm82 expression is a promising metabolic engineering strategy for terpenoids biosynthesis in yeast.

## 5 Conclusion

In summary, this study explored the influence of tRNA m^7^G modification complex, Trm8 and Trm82, on yeast terpenoid metabolism. The overexpression of Trm8 and Trm82 significantly promoted the biosynthesis of squalene in wild type yeast strain. This complex upregulated the expression levels of genes involved in amino acid synthesis, glycolysis, and tricarboxylic acid cycle. Further investigation demonstrated that Trm8/Trm82 increased the production of squalene and lupeol in engineered yeast. These results demonstrated that the overexpression of Trm8/Trm82 could be served as a new metabolic engineering strategy for the bioproduction of terpenoids in yeast.

## Data Availability

The original contributions presented in the study are included in the article, further inquiries can be directed to the corresponding author. The transcriptome data have been submitted to the NCBI Sequence Read Archive (SRA) database under the accession code PRJNA1179691.
