# Supplementary material for: Overexpression of tRNA m7G modification methyltransferase complex promotes the biosynthesis of triterpene in yeast
Source: Front Microbiol. 2025 Mar 31;16:1557443. doi: 10.3389/fmicb.2025.1557443 (PMC11996079; doi:10.3389/fmicb.2025.1557443)
Supplement: Supplementary file 1 [file Data_Sheet_1.docx]

Supplementary Material

# Supplementary Figures

**Supplementary Figure 1.** The verification of expression of Trm8 and Trm82 using RT-qPCR and western blotting (WB) in strain MY01 compared with the control strain. (A) and (B) The RT-qPCR result of *TRM8* and *TRM82*. (C) The WB result of Trm8 and Trm82. (D) and (E) The quantitative data of WB result in (C). The asterisks indicate significant differences (***p* < 0.01, **p* < 0.05).

**Supplementary Figure 2.** The growth curve of CEN.PK2-1C and strain MY01.

**Supplementary Figure 3.** The standard curve of squalene

**Supplementary Figure 4.** The mass spectra of squalene in different strains. (A) squalene standard, (B) MY01 strain, and (C) CEN.PK2-1C.

**Supplementary Figure 5.** The squalene production in strains with deletion of *TRM8*, *TRM82*, and *TRM8*/*TRM82*. The asterisks indicate significant differences (***p* < 0.01, **p* < 0.05).

**Supplementary Figure 6.** The WB result of Hxk1, Pfk2, Eno1 and Cdc19 in glycolysis. The asterisks indicate significant differences (***p* < 0.01, **p* < 0.05).

**Supplementary Figure 7.** The squalene production in strains with HXK1-, ENO1-, PFK2- and CDC19-overexpression. The asterisks indicate significant differences (***p* < 0.01, **p* < 0.05).

**Supplementary Figure 8.** The mass spectra of lupeol. (A) lupeol standard, (B) GgLUS-transformed yeast strain.

**Supplementary Figure 9.** The standard curve of lupeol.

**Supplementary Figure 10.** The metabolic circuit diagrams of effect of Trm8/Trm82 overexpression on squalene production.

# Supplementary Tables

**Supplementary Table S1.** **Primers used in plasmid and strain construction**

| Primer Name | Sequences (5' to 3') |
| --- | --- |
| PGK1p-F | TCCATCGATACTAGTGCGGCCGCTTGTTTTATATTTGTTGTAAAAAGTAGATAATTACTTCC |
| PGK1p-R | AGCTATGGTGTGTGCGGCCGGCCTGGAAGTACCTTCAAAGAATGGGGTCTTATC |
| TRM8-F | AGAATTGTTAATTAAGAGCTCTTACAATATGGCTGGCGTTGGTAATCT |
| TRM8-R | ACAAATATAAAACAAGCGGCCGCATGAAAGCCAAGCCACTAAGCCAAG |
| KILEU-TRM8-F1 | GATCCAGGAGGATGGACATGTTAGATAGAACTTCGTACGCTGCAGGTCGACAA |
| KILEU-TRM8-R1 | ATGAATGACGCTCATGCGATCGCGTCGTATTACGGATCCTTGTAATTAAAACTTAGATTAG |
| KILEU-TRM8-F2 | TACAAGATTACTGATATTTTGATCCAGGAGGATGGACATGTTAGATAGAA |
| KILEU-TRM8-R2 | ATGAGCGTCATTCATCCTTTGCAGAATTTACTCACCTCTC |
| TEF1p-F | ACTTCCAGGCCGGCCGCACACACCATAGCTTCAAAATGTTTCTAC |
| TEF1p-R | TCGTATTACGGATCCTTGTAATTAAAACTTAGATTAGATTGCTATGCTTTCTTT |
| EfMvaS-F | AAGGAGAAAAAACCCCGGATCCATGACCATTGGTATTGATAAAATTTCTTTTTTTGTG |
| EfMvaS-R | AGTGAGTCGTATTACTTAGTTACGATAAGAGCGAACGGTGTTGT |
| EfMvaE-F | GCCAAGCTTGGTACCGAGCTCATGAAAACCGTAGTTATTATTGATGCACTGC |
| EfMvaE-R | AAGGAGAAAAAACCCCGGATCCATGACCATTGGTATTGATAAAATTTCTTTTTTTGTG |
| KITRP-EfMvaS-EfMvaE-F1 | CAACGCCTAAGGAAACTCGTCATATTCTACCTTCGTACGCTGCAGGTCGACAAC |
| KITRP-EfMvaS-EfMvaE-R1 | GAAGCTTGATCGGCTTTTATTCTTTCCTGCCGATTCATTAATGCAGCTGGAT |
| KITRP-EfMvaE-EfMvaS-F2 | GTGAGAAACTCCTACATCAACGCCTAAGGAAACTCGTCATATTCTACCTT |
| KITRP-EfMvaE-EfMvaS-R2 | GCTCCAGTTGTTACCTGAGAATGAAGCTTGATCGGCTTTTATTCTTTCCT |
| ERG8-F | GTTAATTAAGAGCTCTTATTTATCAAGATAAGTTTCCGGATCTTTTTCTT |
| ERG8-R | AGGATGACGACGATAAGATCTATGTCAGAGTTGAGAGCCTTCAGTGC |
| ERG12-F | AGGAGAAAAAACCCCGGATCCATGTCATTACCGTTCTTAACTTCTGCACC |
| ERG12-R | AGTGAGTCGTATTACTTATGAAGTCCATGGTAAATTCGTGTTTCC |
| KIHIS-ERG8-ERG12-F1 | GCAGGAAACGAAGATAAATCATGTCGACTTCGTACGCTGCAGGTCGACAAC |
| KIHIS-ERG8-ERG12-R1 | TAGTTTTGCTGGCCGCATCTTCTCCTTCGAGCGTCCCAAAACCTTCT |
| KIHIS-ERG8-ERG12-F2 | ACAGAACAAAAACCTGCAGGAAACGAAGATAAATCATGTCGA |
| KIHIS-ERG8-ERG12-R2 | TATAATACAGTTTTTTAGTTTTGCTGGCCGCATCTTCTC |
| IDI1-F | TCCATCGATACTAGTTTATAGCATTCTATGAATTTGCCTGTCATTTTC |
| IDI1-R | AACCCTCACTAAAGGGCGGCCGCATGACTGCCGACAACAATAGTATGCCC |
| ERG19-F | AGGAGAAAAAACCCCGGATCCATGACCGTTTACACAGCATCCGTTACC |
| ERG19-R | AGTGAGTCGTATTACTTATTCCTTTGGTAGACCAGTCTTTGCGT |
| GAL10p-ERG10-F | ATACCTCTATACTTTAACGTCAAGGAGAAAAAACC |
| GAL10p-ERG10-R | CCTTTAGTGAGGGTTGAATTTTCAAAAATTCTTACTTTTTTTTTGGATG |
| ERG10-F | AACCCTCACTAAAGGGAGGAACAAGCGCTTCCATTAAGAGC |
| ERG10-R | AGAATTGTTAATTAACGCAGAACGTAATCATATCTTTTCAATGAC |
| ERG10-ADH1t-F | GAAATTTACGAAAAGATGGAAAAGGGTCAAATCGTTGGTAGATAC |
| ERG10-ADH1t-R | ACGCCAGCTGAATTGGAGCGACCTCATGCTATACCTGAGAAAG |
| 308a-gDNA-F: | GAAAGATAAATGATCCACTTGTCAAACAGAATATAGTTTTAGAGCTAGAAATAGCAAGTT |
| 308a-gDNA-R: | CATTTTGAAGCTATGTGCGCAAGCCCGGAATCG |
| SmFPS-F | CGACTCTCCGTTCAGATTCGCCATGAATTTTCAAAAAT |
| SmFPS-R | CCTTTAGTGAGGGTTGAATTCTTATTTCTGCCTCTTGTATATCTTGCCCAAG |
| AtSQS2-F | AAGGAGAAAAAACCCCGGATCCATGGGAAGCTTGAGTACGATTTTGAGAC |
| AtSQS2-R | GTTGTATATCTCAAAGCAAACCAATGTAAGTGAGTAATACGACTCACT |
| KIURA-SmFPS-AtSQS2-F1 | CATTAAAGAGAAATAACAATATTATAAAAAGCGCTTAAACTTCGTACGCTGCAGGTCGACA |
| KIURA-SmFPS-AtSQS2-R1 | CGCATATTTTTTTAACGATTTAAAATCATTAGTTTATCTTCGAGCGTCCCAAAACCTTCTC |
| KIURA-SmFPS-AtSQS2-F2 | TACTAACCTTTCATTAAAGAGAAATAACAATATTATAAAAAGCGCTTAAA |
| KIURA-SmFPS-AtSQS2-R2 | CTGTGTTCGATCCACAGAATTCGCATATTTTTTTAACGATTTAAAATCATTAGTTTAT |
| GgLUS1-F | AGGAGAAAAAACCCCGGATCCATGTGGAAGCTGAAGATAGGAGAAGGAG |
| GgLUS1-R | GTTAGAGCGGATCTTAGCTAGCTCAATAACTGTGAGCACACAAGACTTGG |

**Supplementary Table S2.** **Primers used in qRT-PCR**

| Primer Name | Sequences (5' to 3') |
| --- | --- |
| qUBC6-F | CATCTCGTTGGAGGAAATTCTAGACCCT |
| qUBC6-R | TAGCGATACCAATATAAACCATTGAAGAACTATCA |
| qHXK1-F | CCCAGCAAGAATCGAGGATGATCC |
| qHXK1-R | GCAGCTCTGGTACCGATCAATTCACA |
| qPGI1-F | GGTTGGTAAGGATGAAGAACAAGTTAAGGC |
| qPGI1-R | GTAGGCAATCAAAGCACCCAAAGTAGC |
| qPFK2-F | CGATGGCAGATTTGACGCTAAGCC |
| qPFK2-R | CAGCAATGGCAGCTTGGTTGTCTT |
| qTDH1-F | CGGTAAGGTCTTGCCAGAATTGCA |
| qTDH1-R | CGGCAGCCTTAACAGCCTTCTTG |
| qTDH3-F | GACTGTTGACGGTCCATCCCACAA |
| qTDH3-R | GCCATACCGGTCAACTTACCTTGCA |
| qENO1-F | CGGTACCTTGTCTGAATCCATCAAGGC |
| qENO1-R | GTCTTGATTTGACCAGTTCTCAAACCGA |
| qENO2-F | GCTGACATGTACCACTCCTTGATGAAGAG |
| qENO2-R | GGTTGGTGACAGTCAAGTCATCAGCA |
| qCDC19-F | GACCAAACTGTCCAATCATCTTGGTTACC |
| qCDC19-R | CCGAAGTTGATACGGGCTTCAACAT |
| qPCR-RDN25-1-F | TTGCGGCTGTCTGATCAGGCAT |
| qPCR-RDN25-1-R | CACAAGGACGCCTTATTCGTATCCATC |

**Supplementary Table S3.** **The linkage of transcriptome alterations and squalene/lupeol production**

| Gene name | log2FoldChange of MY01 to control strain | Squalene production in control strain | Squalene production in MY01 | Fold increase of squalene |
| --- | --- | --- | --- | --- |
| HXK1 | 0.489122832 | 1.53 ± 0.41 mg/L | 12.52 ± 0.22 mg/L | 8.2 |
| PGI1 | 0.405693642 |  |  |  |
| PFK2 | 0.298695764 |  |  |  |
| TDH1 | 0.488716788 |  |  |  |
| TDH3 | 0.797902722 |  |  |  |
| PGK1 | 0.55350299 |  |  |  |
| ENO1 | 0.655972183 |  |  |  |
| ENO2 | 0.762852803 |  |  |  |
| CDC19 | 0.225195378 |  |  |  |
